# Should more senior workers be better citizens? Expectations of helping and civic virtue related to seniority

**DOI:** 10.1186/s40064-016-3097-1

**Published:** 2016-09-29

**Authors:** Young-Hee Kang, Ann Marie Ryan

**Affiliations:** 1 Keimyung University, 1095 Dalgubeol-daero, Dalseo-Gu, Daegu, 42601 Republic of Korea; 2 Department of Psychology, Michigan State University, East Lansing, MI 48823 USA

**Keywords:** Altruism, Civic virtue, Courtesy, Expectations, Interpersonal citizenship behavior, Organizational seniority

## Abstract

**Background:**

Organizational citizenship behavior, or extra-role behavior, refers to voluntarily going beyond job task requirements. This study aims to provide a new lens to citizenship behaviors by specifically exploring different expectations of citizenship behaviors related to employees’ demography and suggesting how such expectations might shape employees’ citizenship behaviors.

**Results:**

Using a cross-national sample of 469 workers, interpersonal and helping and civic virtue were more likely to be regarded as in-role behaviors for more senior than for junior employees. On the other hand, results indicate that expectations of courtesy are unrelated to seniority.

**Conclusions:**

By exploring expectations of promotive citizenship behaviors, this study contributes to expanding the OCB literature focused on motives for citizenship behaviors. Findings from this study indicate that there are some significant patterns of expectations related to employees’ seniority. Also, the findings call on managers to set clear boundaries of in- and extra-role behaviors.

## Background

Organizational citizenship behaviors (OCB), labeled as extra-role behaviors or contextual performance, refer to organizational members’ interpersonal and volitional behaviors beyond job-specific task performance (Borman and Motowidlo 1993; Organ 1988). For the last 30 years, OCBs have drawn researchers’ attention because these behaviors are recognized as contributing to organizational effectiveness (Allen et al. 2015; Hart et al. 2016; LePine et al. 2002; Organ 1988; Organ et al. 2006, 2011). As OCB is defined as “voluntary” behaviors of individual members, the majority of OCB research has explored individual differences that affect OCB performance such as personality and psychological attitudes, including organizational commitment, job satisfaction, perceived organizational support, and procedural justice (e.g., Cichy et al. 2009; Podsakoff et al. 2000; Tepper and Taylor 2003; Wagner and Rush 2000), as well as job/role perceptions (e.g., McAllister et al. 2007; Moorman and Blakely 1995).

Organ et al. (2006: 143) mentioned that employees may feel that certain behaviors are expected as part of the job although they believe that the behaviors are beyond the formal job requirement. Also, some scholars have suggested that certain expectations of citizenship behavior may exist in the organization (e.g., Hui et al. 2004; Kidder and McLean Parks 2001; Organ et al. 2006; Vigoda-Gadot 2006, 2007). Unlike research which argues that OCB is voluntary and driven by personal attributes (e.g., Ilies et al. 2006), these researchers suggest that OCB can also refer to involuntary behaviors that are implicitly forced by observers’—coworkers’—expectations (e.g., Hui et al. 2004; Van Dyne et al. 1994) and pressure (e.g., Banki 2010; Bolino et al. 2010; Vigoda-Gadot 2007). That is, workers are likely to sense or recognize their coworkers’ expectations of citizenship behaviors. Despite this recognition, expectations of citizenship behaviors have not been a focus of empirical investigation. This study aims to provide a new lens to citizenship behaviors by specifically exploring different expectations of citizenship behaviors related to employees’ demography and suggesting how such expectations might shape employees’ citizenship behaviors.

An individual’s demographics influence how others perceive and treat them (Tajfel and Turner 1986). In organizational contexts, seniority is a demographic that influences expectations of others (Krings et al. 2011). However, little citizenship behavior research has paid attention to organizational seniority (e.g., Kang 2005; Ng and Feldman 2010), despite meta-analytic work showing the relationship of age to organizational citizenship behaviors (Ng and Feldman 2008). The primary purpose of this study is to examine whether expectations of OCBs are linked to seniority.

In the current study, we specifically focus on expectations of two types of OCBs that are *promotive* (i.e., focused on enhancing organizational effectiveness rather than protecting the stability of operations; Marinova et al. 2010; Podsakoff et al. 2014): interpersonal citizenship behaviors and civic virtue. OCB scholars have suggested the need for a focus on identifying predictors of different types of OCB (e.g., Bowler and Brass 2006; Podsakoff et al. 2000; Settoon and Mossholder 2002). According to McAllister et al. (2007: 1201), interpersonal citizenship behavior, an exemplar of an *affiliative* OCB (Van Dyne and LePine 1998), is a significant predictor of individual and group productivity as well as organizational performance (Podsakoff et al. 2000). Civic virtue refers to responsible participation (Graham 1991, 2000), which involves individuals’ initiative and active participation (Graham and Van Dyne 2006). This OCB domain contains both affiliative and c*hallenging* behaviors (Graham and Van Dyne 2006; McAllister et al. 2007; Morrison and Phelps 1999; Van Dyne et al. 1995). Although challenging OCBs have been less studied than affiliative OCBs, many scholars note that challenging OCBs are necessary to obtain competitive advantages (e.g., Choi 2007; Graham and Van Dyne 2006; LePine and Van Dyne 1998, 2001; Van Dyne and LePine 1998). Both affiliative and challenging OCBs can be focused on promoting organizational effectiveness.

The current research attempts to identify predictors of different types of OCBs by investigating whether different expectations of interpersonal helping and civic virtue are related to seniority. In the pages that follow, we provide a summary of research on interpersonal citizenship behavior and civic virtue. We explicate different expectations for these OCBs related to employees’ seniority. We then examine these hypothesized relations using a sample of employees from global firms.

## Promotive citizenship behaviors: interpersonal citizenship and civic virtue

Van Dyne et al. (1995) defined *promotive* OCBs as those which are proactive and positively intended to enhance the organization (Choi 2007: 469). Interpersonal citizenship is a promotive–affiliative OCB in that it has positive effects on performance by lubricating interpersonal relationships in formally structured settings. In contrast, civic virtue contains both affiliative and challenging citizenship behaviors, as it is positively related to performance by making constructive suggestions on work-related issues (Choi 2007).

### Interpersonal citizenship behavior

As interpersonal citizenship behavior involves helping and cooperating with others (Borman and Motowidlo 1993), and contributes to organizational effectiveness by solidifying and preserving interpersonal relationships, (Podsakoff et al. 2000; Van Dyne et al. 1995), the majority of empirical focus has been on this type of OCB (Choi 2007; McAllister et al. 2007). Scholars have used a variety of labels for these affiliative behaviors, such as altruism (Organ 1988; Smith et al. 1983), OCB-I (Williams and Anderson 1991), interpersonal helping (Graham 1991; Moorman and Blakely 1995), helping coworkers (George and Brief 1992; George and Jones 1997), helping behavior (Podsakoff et al. 2000), helping and cooperating with others (Borman and Motowidlo 1993, 1997), and interpersonal facilitation (Van Scotter and Motowidlo 1996). We do not intend to make distinctions among these concepts but rather to focus more on expectations of interpersonal citizenship behavior more broadly.

### Civic virtue

Civic virtue refers to discretionary behaviors associated with responsible participation (Graham 1986) or engagement in the organization (Podsakoff et al. 2000). These OCBs contribute to organizational effectiveness by providing suggestions for improvement in operations (Choi 2007; LePine and Van Dyne 1998; Van Dyne and LePine 1998). Previous research has also provided evidence that this behavior is a significant antecedent for predicting task performance (e.g., Choi 2007; LePine and Van Dyne 2001; Van Dyne and LePine 1998). Graham and Van Dyne (2006: 91) noted that civic virtue “can range along a continuum from more affiliative (i.e., cooperative, gathering information) to more challenging” (i.e., change-oriented, voice, exercising influence). Affiliative–promotive civic virtue behaviors include attending and participating in meetings and staying informed about the organization; the challenging-promotive aspect of civic virtue includes offering suggestions for organizational improvement (Graham and Van Dyne 2006: 92). Therefore, the current study uses a conceptualization of civic virtue that includes both affiliative and challenging aspects of civic virtue.

## Relations of Social Categorization Theory to expectations of citizenship behaviors

According to Social Categorization Theory (Turner 1987), people are likely to use demographics such as gender, age, race, and education in classifying both themselves and others into social categories (Riordan and Shore 1997: 343; Tajfel and Turner 1986; Tsui et al. 1992). Social categories of a target person play an important role in perceiving stereotypes about his/her likely attitudes, beliefs, and action (Tsui and Gutek 1999: 48) and demography is often used in inferring behaviors and values of a certain social group (Elizabeth and Margaret 2005: 41). Based on demographic factors, people may perceive behaviors as appropriate, inappropriate, or required for themselves and others.

Social Categorization Theory suggests that demographic-related role expectations, in this instance age-related stereotypes, can influence what observers expect as part of one’s role in organizational settings. Age is one of the most salient social and cultural dimensions (Settersten and Mayer 1997: 242). According to Hagestad and Neugarten (1985), age itself means a role that is related to certain expectations for behavior (Wood and Roberts 2006: 1494). There are age-differences in roles that individuals are expected to fulfill in organizations (Lawrence 1996; Wood 1971), desirable behaviors vary across age groups (Wood and Roberts 2006), and age-related stereotypes for behavior exist in organizations (Lawrence 1996; Perry and Parlamis 2006).

In the current study, age norms and age expectations are related to social age rather than just biological age and psychological age. According to Birren and Cunningham (1985: 8), there are three types of age: (1) biological age, which means chronological age; (2) psychological age, which refers to ages associated with capacities of adapting to changing demands; and (3) social age, which involves in the age-graded behavior of individuals expected by their particular society or culture. Also, in this study, age norms would include both prescriptive and proscriptive age norms that refer to shared expectations about what behavior is appropriate or inappropriate for a certain age group (Settersten and Mayer 1997: 242).

In work settings, one’s behaviors can be influenced by expectations of the required or appropriate behaviors related to his/her seniority. Such expectations will give employees information with respect to “what they should do” or “what they should not do” (Kidder and McLean Parks 2001: 940). Potential recipients of OCBs (i.e., coworkers) share certain perceptions of role breadth related to a target individual’s seniority, and such perceptions will lead to expectations of in- and extra-role performance.

### Expectations of interpersonal citizenship behavior related to seniority

According to Podsakoff et al. (2000: 516–517), interpersonal citizenship behaviors include altruism and courtesy. Altruism is defined as helping others in task-related needs, such as giving newcomers orientations, sharing personal possessions with coworkers, and helping coworkers with heavy workloads (Konovsky and Organ 1996). Courtesy refers to helping others by taking steps to prevent the occurrence of problems such as informing others before taking important actions and trying to avoid creating problems for others (Konovsky and Organ 1996).

As more senior employees have greater organizational tenure and life experience, they may be expected to have more behavioral choices in the workplace beyond simply the norm of reciprocity (Caspi and Bem 1990; Kanungo and Conger 1993). Also, more senior workers are also perceived to have greater interpersonal skills and more useful experience, and be more loyal (Smith 2001) and agreeable (Chan et al. 2012). Cross-cultural studies have provided evidence that more senior persons are likely to be kind or benevolent (Giles et al. 2012: 359). In this light, more senior employees could be expected to give more junior colleagues advice or tips about socialization in organizations. Based on the age norm literature, Wood (1971) suggested that emotional support is considered to be a role requirement for senior employees. This does not mean that more junior employees would not be altruistic. Rather, altruistic behaviors are more likely to be perceived as in-role behaviors for senior rather than junior employees.

Similarly, Ng and Feldman’s (2008: 403) meta-analysis found that more senior employees are likely to be “good citizens and control their emotions at work.” Another meta-analysis by Ng and Feldman (2010), which deals with relationships between organizational tenure and job performance, showed that higher tenure is likely to be positively associated with interpersonal citizenship. Based on such a finding, Ng and Feldman (2010: 1245) mentioned that relative organizational tenure (years of tenure relative to colleagues) may influence employees’ perceptions of demonstrating interpersonal citizenship behavior: for instance, employees with relatively long tenure are likely to feel more responsible for helping out their colleagues with short tenure. Thus, we hypothesize:

#### **Hypothesis 1**

Altruism is more likely to be regarded as in-role behavior for more senior than junior employees.

*Courtesy* involves behaviors which contribute to preventing an individual from getting into trouble with others (Organ 1988; Podsakoff et al. 1990). According to Organ et al. (2006: 24), the core of courtesy citizenship is to “avoid practices that make other people’s work harder.” Ng and Feldman (2010: 1243) asserted that compared to short-tenured employees, long-tenured employees may be less likely to incur costs from aggressive or conflictive behaviors than their short-tenured coworkers as such behaviors are more likely to have negative effects on short-tenured employees’ job security or promotional opportunities. The linkage of courtesy behaviors to expectations of junior employees is also supported by literature on communication. For example, Communication Accommodation Theory (CAT: Giles et al. 1991) suggests that employees’ communicative behaviors are influenced by social stereotypes related to age. McCann and Giles (2006) found that younger employees are likely to accommodate when communicating with more senior employees because of obligations to be respectful, whereas those who are more senior are less likely to accommodate in communication. This implies that while all employees may show courtesy to one another, junior employees may be expected to show courtesy behaviors more so than senior employees. Thus, we hypothesize:

#### **Hypothesis 2**

Courtesy is more likely to be regarded as in-role behavior for more junior than senior employees.

#### Expectations of civic virtue related to seniority

As mentioned above, civic virtue citizenship includes challenging behaviors (Graham and Van Dyne 2006; Kidder and McLean Parks 2001). Compared to more junior employees, senior employees are likely to have more work experience and may feel less constrained in voicing views for change. More junior employees are usually younger and have lower level positions, and therefore have less power than more senior employees. In this regard, Conway (1996) argued that civic virtue is expected to be more in-role behavior for managers than for non-managers.

In addition, Ng and Feldman (2010) addressed that longer tenure may lead to high levels of knowledge about routines of the organization and organizational tenure has been considered a proxy for job-related knowledge (Ng and Feldman 2010). Human capital theory implies that longer tenure in an organization contributes to enhancing knowledge about the organizational goals, the formal power structures, and work processes or procedures in the organization (Ng and Feldman 2010). In terms of suggesting ideas of work or organizational changes, more senior employees may be more likely to voice ideas than juniors because of their greater knowledge of organizational workings and history (Ng and Feldman 2010: 1224). Ng and Feldman’s (2013) meta-analysis provided evidence that longer-tenured and senior employees are more likely to engage in innovation-related behaviors than short-tenured and junior employees. Thus, others may come to expect senior employees to exhibit more civic virtue citizenship than junior employees.

##### **Hypothesis 3**

Civic virtue is more likely to be regarded as in-role behavior for more senior than junior employees.

## Methods

### Sample and procedure

Participants were recruited from white-collar employees of both South Korean and US sites of a large South Korean multinational automobile company and a bank. In particular, we collected the automobile data from diverse business divisions (operating, sales & marketing, R&D, and planning support) in South Korea and the US headquarter and the US R&D division. On the other hand, the bank data was collected from employees of three US branches because the HR department of South Korea denied participating in the survey. We used a paper–pencil survey and participants were requested to complete the 20-min long questionnaire during working hours. The questionnaire had two language versions, Korean and English. 745 questionnaires were distributed and 469 questionnaires were returned completed for an overall response rate of 63 %. As this sample had missing values for less than 10 % of the responses, we used multiple imputation (number of imputations = 5) to deal with missingness. There were 371 (79.1 %) participants from the automobile company and 98 (21.9 %) from the bank. 374 (80.2 %) were males and the age of respondents ranged from 21 to 59 with a mean of 37.4 (*SD* = 7.41). The average tenure was 114.6 months (*SD* = 85.1). 11 participants (2.4 %) had a high school or lower degree, 35 (7.6 %) had a 2-year college degree, and 416 (90 %) had a 4-year university or higher degree. Most of the participants (98.3 %) were regular full-time workers. 54 (11.6 %) were supervisors and there were 381 (81.2 %) South Koreans, 76 (16.2 %) Americans, and 12 (2.6 %) others.

### Measures

As the survey questionnaires were initially written in English and translated into Korean, we adopted the procedure recommended by Brislin (1986). Translators did not know the proposed study’s hypotheses, and two bilinguals separately translated the survey from English to Korean and from Korean to English. A third bilingual individual then translated the Korean survey back to English. Then, the Korean survey was pre-tested with around 30 employees of a large Korean construction firm. Participants were asked to give comments on any item that was ambiguous or difficult to understand. Their comments resulted in only minor changes.

#### Expectations of OCB

*Expectations of OCB* (EOCB) was measured by the extent to which a particular OCB is considered by workers to be in-role behavior for their colleagues. To measure EOCB, we modified Konovsky and Organ (1996)’s 12 items for altruism (5 items), courtesy (4 items), and civic virtue (3 items). We provided questions on *expectations of OCB* for senior and junior employees separately and the responses to questions on seniors and juniors were dummy-coded (0 = *juniors*, 1 = *seniors*). As age, tenure, and job position are confounded with seniority (Gosseries 2004), we provided the definition of senior and junior employees to respondents. In this study, tenure in the current organization was used as the operationalization of seniority. Seniors (or juniors) were defined as employees who have longer (or less) tenure in the current organization. To measure expectations, we added the phrase “I think that seniors (juniors) should” to each item of OCB. For example, one item was “I think that senior (juniors) coworkers should help coworkers who have been absent.” The ratings were on a 7-point Likert scale ranging from (1) *strongly disagree* to (7) *strongly agree*. Higher scores on the expectations of seniors’ citizenship and those of juniors’ indicate a higher extent of expectations of OCB attached to senior employees and juniors, respectively.

Because the EOCB measure was adapted for this study, we evaluated the factor structure. We first conducted exploratory factor analyses (EFAs) with principal axis factor (PAF) and oblique promax rotation on the overall sample. EOCB measures were modified from Konovsky and Organ’s (1996) OCB scale. PAF is recommended when variables are measured with scales that have not been tested statistically (Dekas 2010). Promax was adopted in that it is good choice when OCB constructs are correlated (Costello and Osborne 2005). Also, factor loadings less than .35 were suppressed because a cut-off for selecting items ranges from .30 to .40 in ERB studies (i.e., Konovsky and Organ 1996; Moorman and Blakely 1995).

As expected, results of the EFA indicated three OCB constructs. The initial factor analysis, which included 12 items of *expectations of OCB*, supported a three-factor model that accounted for 58.29 % of the total variance in *expectations of senior employees’ OCB* and 59.99 % of the total variance in *expectations of junior employees’ OCB* (see Tables 1, 2 for factor loadings). We selected items with factor loadings more than .35 (Floyd and Widaman 1995). As the result of EFAs, both the *expectations of senior employees’ OCB* and *expectations of junior employees’ OCB* scales have three common factors with 12 items of the *expectations of coworkers’ OCB* scale: altruism (5 items), courtesy (4 items), and civic virtue (3 items). The Cronbach’s α of the *expectations of employees’ OCB* dimensions ranged from .70 to .78, yielding reasonable reliabilities (Nunnally 1978).

**Table 1:** Exploratory factor analysis (EFA) results of the overall sample: expectations of OCB for senior coworkers

Factor	Factor 1^a^	Factor 2^b^	Factor 3^c^
Eigenvalue	4.18	1.50	1.32
Percentage of the variance	34.82	12.51	10.97
Help others who have heavy workloads	*.86*		
Help others who have been absent	*.64*		
Help make other workers productive	*.65*		
Help orient new people even though it is not required	*.50*		
Share personal property with others if necessary to help them with their work	*.44*		
Respect the rights and privileges of others			*.42*
Try to avoid creating problems for others			*.71*
Consult with other people who might be affected by their actions or decisions			*.80*
Inform others before taking any important actions			*.55*
Stay informed about developments in the company		*.79*	
Attend and participate in meetings regarding the company		*.82*	
Offer suggestions for ways to improve operations		*.64*	
Cronbach’s α	.72	.78	.72

*N* = 469; Extraction Method: Principal Axis Factoring; Rotation Method: Promax with Kaiser Normalization; Factor loadings (italics) less than .35 are suppressed

^a^Expectations of seniors’ altruism

^b^ Expectations of seniors’ civic virtue

^c^ Expectations of seniors’ courtesy

**Table 2:** Exploratory factor analysis (EFA) results of the overall sample: expectations of OCB for junior coworkers

Factor	Factor 1	Factor 2	Factor 3
Eigenvalue	3.95	1.83	1.30
Percentage of the variance	32.91	15.25	10.84
Help others who have heavy workloads	*.74*		
Help others who have been absent	*.68*		
Help make other workers productive	*.75*		
Help orient new people even though it is not required	*.59*		
Share personal property with others if necessary to help them with their work	*.56*		
Respect the rights and privileges of others		*.51*	
Try to avoid creating problems for others		*.81*	
Consult with other people who might be affected by their actions or decisions		*.69*	
Inform others before taking any important actions		*.41*	
Stay informed about developments in the company			*.76*
Attend and participate in meetings regarding the company			*.81*
Offer suggestions for ways to improve operations			*.63*
Cronbach’s α	.78	.70	.78

*N* = 469; Extraction Method: Principal Axis Factoring; Rotation Method: Promax with Kaiser Normalization; Factor loadings (italics) less than .35 are suppressed

^a^Expectations of juniors’ altruism

^b^ Expectations of juniors’ courtesy

^c^ Expectations of juniors’ civic virtue

Cross-cultural study scholars have recommended assessing measurement invariance before testing hypothesized relationships (e.g., Tsui et al. 2007). Measurement invariance indicates the extent to which a certain measure can be universally applied to different conditions (Drasgow 1984; Horn and McArdle 1992). However, given the number of *expectations of OCB* items and dimensions, the US sample (*n* = 88) is small for testing measurement equivalence of both scales. As results of EFAs show, the *expectations of senior employees’ OCB* and *expectations of junior employees’ OCB* scales had three common factors on the overall sample. Analyses with just the Korean sample provided evidence that both senior and junior measures are equivalent: ∆χ^2^ (11, *N* = 381) = 8.99, *p* = .62, CFI = .90, ΔCFI = 0 (Cheung and Rensvold 2002). This means that quantitative comparison between these measures is defendable. Thus, for testing the hypotheses, it is assumed that the two measures are invariant across junior and senior versions.

#### Control variables

To test the relationships between *expectations of OCB* and variables of interest, we controlled for some variables. First, job position was controlled in that supervisors are likely to perceive OCB as in-role behavior as well as consider OCB performance and task performance to the same extent in performance evaluation (e.g., Borman 1987; MacKenzie et al. 1991; Podsakoff and Mackenzie 1994; Werner 1994). Another control variable is country in that there are differences in perceptions of OCB between countries (e.g., Farh et al. 2004). In this study, country refers to the location of work sites (South Korea and the U.S.) rather than participants’ nationality. Finally, industry served as a control variable in that some studies suggested that there are industrial differences in OCB performance (e.g., Ariani 2012; Chiang and Hsieh 2012; Raub 2008). Thus, control variables are job position (0 = *non*-*supervisor*, 1 = *supervisor*), industry (0 = *manufacturing organization*, 1 = *service organization*), and country (0 = *South Korea*, 1 = *US*).

## Results

Descriptive statistics, missing values, correlations, and reliability (Cronbach’s α) for the South Korean and the US samples are presented in Table 3. There are a couple of points to note. First, variables of interest are significantly correlated with one another in each of the samples as expected. Also, both samples yielded acceptable reliabilities for the items of EOCB (.79 ≤ Cronbach’s *α* ≤ .86).

**Table 3:** Basic statistics and correlations for samples

Variable	*M*	*SD*	1	2	3	4	5	6
*Korean sample*								
1. Altruism^a^	5.55	.76	(.86)					
2. Courtesy^b^	5.74	.69	.45**	(.81)				
3. Civic virtue^c^	5.62	.80	.42*	.41**	(.86)			
4. Seniority^d^	.50	.50	.11**	−.01	.16**	–		
5. Position^e^	.07	.26	.16**	.04	.18**	0	–	
6. Industry^f^	.23	.42	.09	.05	.10**	0	.13**	–
*US sample*								
1. Altruism	4.92	.89	(.80)					
2. Courtesy	6.07	.69	.27**	(.82)				
3. Civic virtue	5.83	.79	.36**	.18*	(.79)			
4. Seniority	.50	.50	.23**	−.09	.25**	–		
5. Position	.30	.46	.20**	.17*	.18*	0	–	
6. Industry	.10	.30	.17*	.02	-.10	0	.11	–

Korean sample (*n*) = 361–381. US sample (*n*) = 78–88. Cronbach’s α appears in parentheses

* *p* < .05. ** *p* < .01

^a^Expectations of altruism

^b^ Expectations of courtesy

^c^ Expectations of civic virtue

^d^ 0 = *junior*, 1 = *senior*

^e^ 0 = *non*-*supervisor*, 1 = *supervisor*

^f^ 0 = *manufacturing*, 1 = *service*

As participants were asked to respond to EOCB measures for senior and junior employees, a repeated measure approach is appropriate. For testing the proposed hypotheses, we conducted repeated measures design with Mixed Model in the statistical software package SPSS 20: seniority (junior, senior) as a within-subjects factor and control variables (job position, industry, and country) as between-subjects factors. Table 4 displays the repeated measures with Mixed Model results.

**Table 4:** Results of repeated measures: type III tests of fixed effects relationships between expectations of OCB and seniority

	Altruism				Courtesy				Civic virtue			
	B	*df*	*t*	η^2e^	B	*df*	*t*	η^2d^	B	*df*	*t*	η^2f^
Intercept	5.451 (.097)	888	56.170***		6.223 (.088)	922	70.416		6.363 (.098)	894	65.030	
Seniority^a^	.212 (.050)	919	4.228***	.016	−.030 (.045)	933	−.667	0	.283 (.050)	924	5.262***	.032
Job position^b^	.407 (.062)	919	4.996***	.023	.152 (.074)	933	2.063*	.005	.425 (.082)	924	5.184***	.027
Industry^c^	.136 (.067)	919	2.191*	.005	.060 (.056)	933	1.075	.001	.098 (.062)	924	1.567	.003
Country^d^	−.687 (.081)	919	−10.226***	.098	.309 (.061)	933	5.092***	.027	.132 (.068)	924	1.994	.003
*Adj. R* ^2^ (*R* ^2^)	.132 (.135)				.037 (.041)				.070 (.074)			

*N* = 469. Unstandardized beta reported; *SE* reported in parenthesis

*** *p* < .001; ** *p* < .01; * *p* < .05

^a^ 0 = *junior*, 1 = *senior*

^b^ 0 = *non*-*supervisor*, 1 = *supervisor*

^c^ 0 = *manufacturing*, 1 = *service*

^d^ 0 = *South Korea*, 1 = *US*

^e,d,f^ Classical eta squared, which is based on the form suggested by Richardson’s (2011: 142): SS_effect_/SS_Total_; however, the interpretation of effect size indices needs caution in that the effect size indices may be easily misinterpreted (Richardson 2011; Levine and Hullett 2002; Olejnik and Algina 2003)

For expectations of altruism, there was a significant effect of an employee’s seniority on coworkers’ expectations, B = .212 (*SE* = .05), *t*(979) = 4.27, *p* < .001, η^2^ = .016, with expectations of senior employees’ altruism receiving higher scores than that of juniors. This provided support for H1, indicating that altruism is more likely to be regarded as seniors’ in-role behaviors than juniors’.

Hypothesis 2 predicted that courtesy is more likely to be regarded as in-role behaviors for junior employees than for senior employees. There was no significant difference in coworkers’ expectations of courtesy for seniors versus junior employees, B = −.030 (*SE* = .045), *t*(933) = −.667, *p* = .505, η^2^ = 0. H2 was not supported, suggesting that courtesy is not more likely to be expected in-role behavior more so for junior than senior employees.

For civic virtue, there was a significant effect for employees’ seniority, B = .283 (*SE* = .050), *t*(924) = 5.626, *p* < 001, η^2^ = .032, with coworkers’ expectations of senior employees receiving higher scores than for junior employees. This provided support for H3, indicating that civic virtue is more likely to be regarded as in-role behavior for senior than for junior employees.

## Discussion

This study provides some exploratory glimpses of what expectations of OCB linked to seniority exist in the organizational setting by testing different expectations of senior employees’ OCB from those of juniors’. Employees’ seniority was found to have significant influence on expectations of OCB: senior employees are likely to be expected to engage more in interpersonal helping (i.e., altruism) and civic virtue. Consistent with the socially responsible norm of benevolence for senior employees (Kanungo and Conger 1993), employees regard altruistic behaviors as senior colleagues’ in-role behaviors more so than for junior employees. Civic virtue is the form of gathering information (e.g., Graham and Van Dyne 2006) and suggesting improvements for the organization (e.g., Graham and Van Dyne 2006; Ng and Feldman 2013) behaviors that might require experience. Therefore, senior employees are expected to engage in civic virtue more so than junior employees. On the other hand, results indicate that expectations of courtesy are unrelated to seniority. Courtesy may be considered in-role for both senior and junior employees in that its scale mean (*M* = 5. 801, *SD* = .703) was higher than that for the altruism (*M* = 5.436, *SD* = .824), *t*(937) = 12.587, *p* < . 001, and civic virtue (*M* = 5.659, *SD* = .80) measures, *t*(937) = 5.177, *p* < .001.

### Contributions

The findings of the present study have implications for research on OCBs. First, the current study suggests the need to continue to expand citizenship behavior research beyond personal (personality, affect, etc.) and situational (job characteristics, social settings, etc.) antecedents of OCB. Often, the existing literature has assumed that whether a person performs a citizenship behavior or not is a personal choice (Banki 2010) with little relation to expectations from other members within the organization. The current research shows the value of considering others’ expectations of citizenship behavior.

Also, the findings from this study provide some understanding of why employees are likely to engage in certain OCBs more than others. Some scholars have provided empirical evidence that employees may engage in OCB due to strong peer pressures or expectations rather than their own good will (e.g., Vigoda-Gadot 2007). For example, “compulsory citizenship behavior” suggested by Vigoda-Gadot (2006, 2007) implies that extra-role behaviors may be performed under pressure from supervisors or management. However, as these studies focused on employees who perform OCBs, they did not provide comprehensive explanations for the process. By incorporating potential OCB beneficiaries’ expectations of OCB, this study provides explanations of motives for such involuntary OCB. Coworkers’ expectations may implicitly impose particular citizenship behaviors on a certain category of workers, which in turn coerces workers into engaging in these behaviors.

In addition, Podsakoff et al. (2000) suggested that further research needs to be conducted on identifying predictors of different types of citizenship behaviors. Although some research has suggested that psychological attitudinal variables such as job satisfaction, organizational commitment, or procedural justice are significant predictors of interpersonal citizenship behavior (e.g., Devece et al. 2016; Tepper and Taylor 2003; Wagner and Rush 2000), other scholars have addressed that such variables are less likely to be a good predictor for explaining variation in the citizenship performance (e.g., Bowler and Brass 2006; Korsgaard et al. 1997). This implies that in order to predict altruism performance, other significant antecedents or correlates of interpersonal helping need to be identified. Therefore, the findings from this study might contribute to gap beyond attitudinal variables as antecedents of affiliative citizenship behaviors.

Results of this study also hold significant implications for practitioners. The findings call on managers to set clear boundaries of in- and extra-role behaviors. Scholars have provided empirical evidence that supervisors take into consideration task performance and citizenship behaviors in performance ratings (e.g., Borman 1987, 2004; Mackenzie et al. 1991; Podsakoff and MacKenzie 1994; Podsakoff et al. 2000). Similarly, peers tend to pay attention to interpersonal citizenship in addition to task performance when rating overall performance (Conway 1999). The results of this study provide support for that supervisors are likely to consider citizenship behavior task performance. As seen in Table 4, compared to employees at the non-supervisory position supervisors relatively have high levels of citizenship behaviors. In particular, supervisors show higher levels of altruism expectations than do non-supervisors (B = .407 (*SE* = .062), *t*(919) = 4.996, *p* < 001, η^2^ = .023). Also, for expectations of courtesy supervisors show higher levels of expectations than non-supervisors, B = .152 (*SE* = .074), *t*(933) = 2.063, *p* < 005, η^2^ = .009. Likewise, supervisors display higher levels of expectations of civic virtue, B = .425 (*SE* = .082), *t*(924) = 5.184, *p* < 001, η^2^ = .027.

As supervisors are potential OCB receivers as well as performance raters, their expectations of OCB related to seniority may affect performance evaluations of senior subordinates. These imply that it is important to clearly define an individual’s duty in organizations where an individual’s compensation is based on individual performance. Also, Welbourne et al. (1998) have addressed that as “performance rating should be largely based on the social roles that employees play in organizations” (as cited in Ng and Feldman 2008: 408), senior employees’ citizenship performance may need to be weighted more heavily than their job-task performance. In this regard, Ng and Feldman (2008) suggest that to reduce age bias in the evaluations, a review of how these behaviors are weighted might be undertaken (Levy and Williams 2004; Perry and Finkelstein 1999).

Another practical implication is that there is industrial difference in expectations of helping, particularly those of altruism. Participants from the bank tend to show higher levels of altruism expectations than those from the manufacturing, B = .136 (*SE* = .067), *t*(919) = 2.191, *p* < 05, η^2^ = .006. Usually the service transactions take place face-to-face in interactions between service agents and customers. Employees in service organizations may go beyond their job duty in order to meet their customers. In particular, service industries that require high levels of service quality are more likely to show higher expectations of coworkers’ helping. This implies that service organizations may need to remove such implicit pressures of helping behaviors which can result in job-related stress or role-overload.

In addition, the findings of this study contribute to international HRM in global business environments. As seen in Table 4, expectations of coworkers’ affiliative behaviors may vary across countries. In particular, employees from the Korean sites are more likely to show high levels of altruism expectations than are those from the US sites (B = −.687 (*SE* = .081), *t*(919) = −10.226, *p* < 001, η^2^ = .098). Employees from the US sites have higher levels of expectations for courtesy than those of the Korean sites, B = .309 (*SE* = .061), *t*(933) = 5.092, *p* < 001, η^2^ = .027. This indicates that altruism is more likely to be considered in-role behaviors in Korea while so is courtesy in US contexts. This implies that multinational companies should reduce differences in expectations of in- and extra-role behaviors in the multicultural workplace.

### Limitations

Despite the contributions and strengths, this study has some limitations. First, as the US sample data of the current study was not large enough to test measurement invariance, we assumed that measures of *expectations of OCB* for senior and junior employees were invariant across samples for testing hypotheses. However, for a cross-cultural and comparative study, validating measures across cultures or nations is important (Ryan et al. 1999; Tsui et al. 2007). While the scales had a common factor structure in both the Korean and US sample, more rigorous evidence of equivalence across cultures would be desirable.

Next, for cross-national studies, samples need to be comparable (Brislin et al. 1973). In this study, the Korean sample (*n* = 292) is relatively larger than the US sample (*n* = 127) although some-cross cultural studies used small samples (e.g., Turnipseed and Murkison 2000). Also, the US sample was not equivalent to the South Korean sample in some demographics: age (*p* < .001), tenure (*p* < .001), industry (*p* = .006), and job position (*p* < .001). In particular, the US sites of the participating businesses had a relatively shorter history because the company opened US sites later than those in South Korea. Controlling for location in our analyses addressed this concern in terms of our findings, but is important to consider in future research on expectations of OCBs.

Another limitation of this study involved the characteristics of participants. The majority of participants were male, full-time, and white-collar workers which may limit applicability to a gender-balanced or female-dominant sample. However, we found that there were non-significant gender differences in *expectations of OCB*. Also, this study focused on expectations of white-collar workers, who usually are highly educated. Future research needs to be conducted on more occupationally and educationally diverse samples, particularly as in-role prescriptions differ by occupation. Similarly, the sample of this study was collected from an automobile company for and a bank, which means that the findings from this study may have limited generalizability. Further research should be conducted on samples of various companies and industries.

### Directions for future research

Findings from this study suggest some opportunities for future research on expectations of interpersonal citizenship and civic virtue associated with demographics. Further research needs to be conducted on other demographic factors that may influence expectations of citizenship behaviors such as gender (female vs. male), job position (i.e., supervisor vs. non-supervisor), and employment type (i.e., regular employment vs. irregular employment).

Another possible opportunity for future research is to explore the relationships between potential OCB recipients’ characteristics and their expectations of OCB. Social identity theory suggests that people usually self-categorize through group membership such as “age, gender, organizational affiliation, occupational affiliation, or religious membership” (Ashforth and Mael 1989; Tajfel and Turner 1986; as cited in Todd and Kent 2009: 174). In this light, coworkers’ social identity may influence expectations of the target employee’s citizenship behavior. For example, as females are more likely to engage in helping behaviors than males are (Mesch et al. 2011), helping might be considered females’ in-role behaviors more than males’ (Kidder and McLean Parks 2001).

Further research needs to be conducted within organizational contexts that have different definitions of seniority. For instance, professors with 5 years of tenure are unlikely to be regarded as senior employees, but as employees are likely to move from company to company in the high technology industry, someone with 5 years of tenure might be viewed as quite senior in that setting (Carnoy et al. 1997: 42).

Another promising research focus is on the linkage between *expectations of OCB* and target workers’ seniority based on job tenure. In this study, the choice to focus on organizational seniority was based on literature which indicated that organizational tenure was more influential than job tenure (e.g., Gosseries 2004; Huang et al. 1998). However, for certain workforce segments such as professionals, job tenure might be as critical as organizational tenure in terms of demography.

The current study tested only expectations of task-focused interpersonal helping not including person-focused interpersonal helping. According to Settoon and Mossholder (2002), person- and task-focused interpersonal citizenship behaviors are conceptually distinct and have different antecedents each other. Therefore, future research needs to be conducted on expectations of person-focused helping behaviors, as well as on expectations of other OCBs such as conscientiousness and sportsmanship.

## Conclusions

By exploring expectations of promotive citizenship behaviors, this study contributes to expanding the OCB literature focused on motives for citizenship behaviors. Findings from this study indicate that there are some significant patterns of expectations related to employees’ seniority. Further research on expectations of citizenship behaviors will enrich the scholarship of OCB.
